# Extracellular activity of a bacterial protease associated with reduced phage infectivity

**DOI:** 10.1371/journal.pone.0332566

**Published:** 2026-05-14

**Authors:** Ehud Herbst, Gael Rosen Blechman, Taya Fedorenko, Sarah Melamed, Gil Amitai, Rotem Sorek

**Affiliations:** Department of Molecular Genetics, Weizmann Institute of Science, Rehovot, Israel; Universidad Nacional Autonoma de Mexico Facultad de Quimica, MEXICO

## Abstract

To defend against bacteriophage (phage) infection, bacteria have developed various defense systems, dozens of which were discovered and mechanistically studied recently. To date, almost all defense systems whose mechanisms were deciphered were shown to operate within the bacterial cell. Here we describe a secreted protease from the Actinobacterium *Salinispora mooreana* which, when expressed heterologously in *Streptomyces coelicolor*, reduces titers of two taxonomically related *Siphoviridae* phages. Antiphage effects were maintained when concentrated supernatant from *S. coelicolor* expressing the *Salinispora* protease was added externally to phage-containing medium, in either the presence or absence of bacterial cells, supporting an extracellular mechanism. We further show that phages can escape the antiphage effect of the *Salinispora* protease by mutating a tail-associated protein. The antiphage effect is associated with an increased proportion of phage particles devoid of DNA. Our data suggest antiphage activity of a secreted bacterial protease.

## Introduction

Over the past years, many immune systems that protect bacteria from bacteriophage (phage) infection were discovered and characterized [1–3]. Various mechanisms of defense were described, such as degradation of phage nucleic acids, regulated death of infected cells, inhibition of protein translation and DNA replication, and depletion of essential metabolites [4,5]. Most of the defense systems studied to date operate inside the bacterial cell after the phage has injected its DNA into the cell.

Previous studies have proposed that bacteria might use extracellular proteases to directly inactivate phage particles, but mechanistic studies to directly associate extracellular protease activity to an antiphage effect were limited. Hoque et al showed that incubation of *Vibrio* phages with spent media of *Vibrio cholerae*, exhibiting protease activity, led to decreased phage titers [6]. Castillo et al showed that incubation of phages in filtered supernatants from *Vibrio anguillarum* strains led to a reduction of up to 10^3^-fold in phage titers [7]. This phage titer reduction was partially reversed by EDTA addition, supporting a potential proteolytic function. However, the identity of the putative proteases responsible for the proteolytic function was not determined [7].

Extracellular proteases have diverse roles in bacterial cell biology. For example, extracellular proteases from *Clostridia* are efficient collagenases; they enable these bacteria to colonize their host and are used clinically for treatment of collagen-related diseases [8–10]. *Streptomyces griseus* trypsin, encoded by the *SprT* gene, is an enzyme active at the onset of sporulation [11]. The production of another extracellular protease, with chymotrypsin-like activity in *Streptomyces exfoliatus*, was associated with mycelium formation and suggested to be important for the acquisition of proteinaceous nitrogen sources [12].

In this study, we report the identification of a bacterial gene coding for a putative extracellular protease from the Actinobacterium *Salinispora mooreana*, which exhibits an antiphage activity. *Streptomyces coelicolor* that heterologously expresses this protease can be infected by *Streptomyces* phages, but following replication and prolonged incubation, some phages lose infective titers by up to three orders of magnitude. The effect is specific for two homologous phages among a collection of *S. coelicolor* phages tested. The antiphage effect of the *Salinispora* protease is maintained when supernatant of *S. coelicolor* expressing the *Salinispora* protease is added externally to *S. coelicolor* cells not expressing the *Salinispora* protease, or incubated with phages in the absence of bacterial cells, supporting an extracellular activity of the secreted protease. By isolating mutant phages that can escape the antiphage effect of the *Salinispora* protease we identify a phage gene encoding for a structural protein that is repeatedly mutated in escaper phages. Our electron microscopy data suggest that the antiphage effect of the *Salinispora* protease is associated with counterproductive DNA ejection from affected phages.

## Results

**Fig 1:**
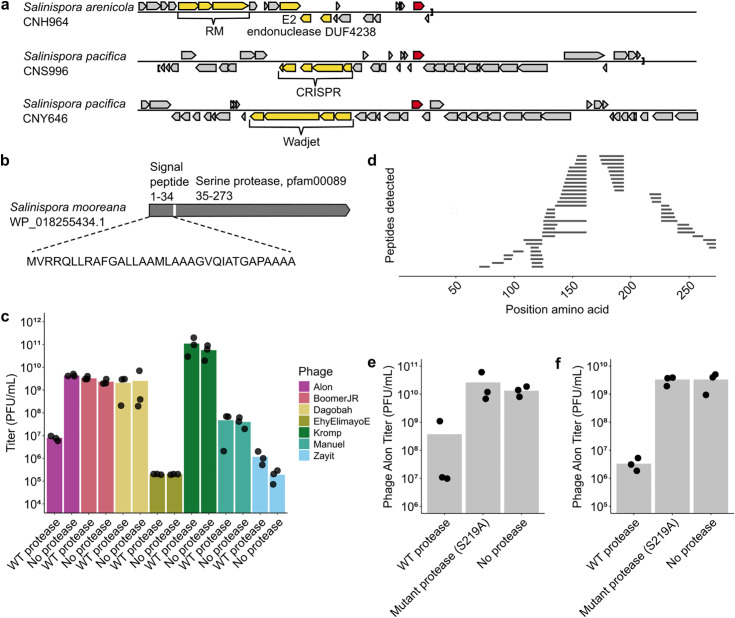
A secreted protease associated with reduced phage titers. **a**. Genomic neighborhoods of a group of predicted secreted proteases (red) in *Salinispora* species. Genes known to be involved in bacterial antiphage defense are in yellow; other genes in grey. **b**. Position of a signal peptide in the *Salinispora* protease as predicted by the SignalP-6.0 software [13]. **c**. Titers of seven phages following infection with *S. coelicolor* expressing the WT *Salinispora* protease or an apramycin resistance gene used as a negative control. Phages were initially added at multiplicity of infection (MOI) of 0.05, and harvested from the culture two days from the onset of infection. Phages were then counted by plating on an indicator strain. Bars show the averages of three biological replicates with individual data point overlaid. **d**. Mass spectrometry (MS) analysis indicates presence of the protease in supernatants. Shown are non-redundant peptides (n = 60) detected using MS. *S. coelicolor* expressing the WT *Salinispora* protease was grown for 46 h, and the supernatant was harvested and filtered. Samples were digested with trypsin and were then subjected to protein MS. **e**. Titers of phage Alon following infection in the presence of protease-containing supernatant. WT or mutant protease-expressing *S. coelicolor* cells or cells not expressing the protease were grown for two days in the absence of phage, and supernatant was collected, filtered and concentrated. This supernatant was added to an infection experiment in liquid culture with *S. coelicolor* cells not expressing the protease and phage Alon at MOI = 0.1. Phages were then harvested from the culture two days after infection. Phage titer counts shown are averages of three biological replicates with individual data point overlaid. **f.** Titers of phage Alon following infection with *S. coelicolor* expressing the WT *Salinispora* protease (WT), a catalytically inactive protease mutant or an apramycin resistance gene instead of the protease as control. Phages were initially added at MOI = 0.1, and harvested from the culture two days from the onset of infection. Bars show the averages of three biological replicates with individual data point overlaid.

While examining putative gene cassettes predicted to encode antiphage pathways in bacteria from the genus *Salinispora*, we noticed a gene coding for a protease that was sometimes present next to various defense systems in these bacteria (Fig 1A). This gene caught our attention because its N-terminus was predicted to harbor a signal peptide, suggesting that it encodes for a secreted protease with extracellular proteolytic activity (Fig 1B). We decided to test the protease-encoding gene from *Salinispora mooreana* for antiphage defense. We chose *S. coelicolor* as the heterologous host to express this gene, because of its frequent use as a heterologous host for expressing genes derived from species of the *Actinomycetota* phylum, and because a variety of phages that infect this species were described [14–17].

To test whether the *Salinispora* protease has an antiphage effect, we performed infection experiments in liquid culture. *S. coelicolor* expressing the *Salinispora* protease, as well as control *S. coelicolor* not expressing the protease, were subjected to infection by a set of seven diverse *S. coelicolor* phages, some of which obtained from culture collections while others were isolated by us. We followed the dynamics of phage replication by taking samples from the liquid culture two days following phage addition, and used plaque assays on an indicator protease-less *S. coelicolor* to count phages. Following prolonged incubation for two days, the infective titers of phage Alon, but not any of the other phages, was reduced by three orders of magnitude when infecting the protease-expressing strain (Fig 1C).

The *Salinispora* protease was predicted by the SignalP-6.0 software to encode an N-terminal twin arginine translocation signal peptide of 34 amino acids with 99.9% likelihood [13]. We verified by protein mass spectrometry (MS) that the *Salinispora* protease is indeed found in the supernatant from a culture of *S. coelicolor* expressing the *Salinispora* protease (Fig 1D, S1 Table in S17 File). Peptides spanning the N-terminal signal peptide were depleted in these data, further supporting the hypothesis that this protease is secreted and the signal peptide is cleaved in the process (Fig 1D).

To test the hypothesis that the *Salinispora* protease exerts an antiphage activity extracellularly, we added concentrated supernatants from *S. coelicolor* expressing the *Salinispora* protease to media in which we performed infection experiments with phage Alon and *S. coelicolor* not expressing the protease. The results of this experiment demonstrated supernatant-dependent reduction in titers of phage Alon (Fig 1E).

To further substantiate that the proteolytic activity of the *Salinispora* protease is responsible for phage titer reduction, we substituted serine residue 219 in the protease, predicted to participate in the catalytic triad of the active site [18–20], to alanine. This residue is found in the highly conserved GDSGGP motif of the catalytic triad [20]. Indeed, our data show that phage Alon infecting *S. coelicolor* cells that express the catalytically inactive S219A variant does not exhibit titer reduction (Fig 1F). In further support of this result, adding concentrated supernatant from *S. coelicolor* expressing the catalytically inactive S219A protease to media where infection experiments where performed with phage Alon and *S. coelicolor* lacking the protease, did not lead to phage titer reduction (Fig 1E). We verified that the S219A catalytically inactive *Salinispora* protease mutant expresses in *S. coelicolor* (Fig S1 in S1 File).

**Fig 2:**
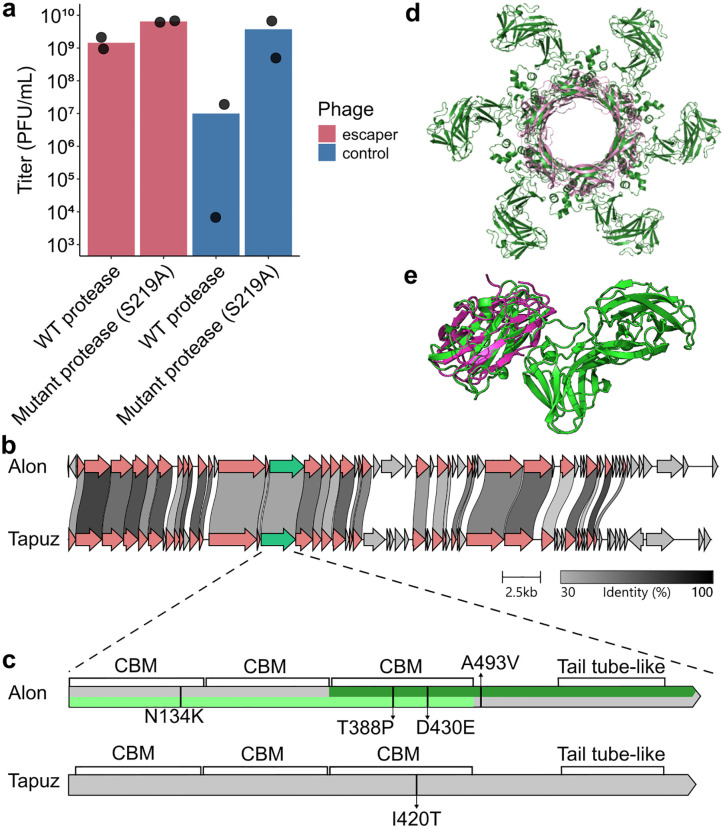
Phages escape the effect of the protease via mutations in a tail-associated gene. **a.** Phage Alon variants selected on bacteria expressing the WT *Salinispora* protease (red) or catalytically inactive protease (blue). Results shown for a representative escaper mutant and control are averages of two biological replicates (two separately isolated escaper phages with the same genotype) with individual datapoints overlaid. Data for all phage Alon mutants that were isolated and sequenced are presented in Fig S3 and Table S2 in S1 File. **b.** Genome comparison of phage Alon and phage Tapuz. Amino acid sequence similarity in the range of 30%−100% is marked by grey shading. The gene mutated in all escapers is marked in green. Other genes with over 30% identity between the two phages are marked in red. Visualized using clinker [22]. **c.** Position of point mutations on the phage tail-associated protein, identified in phage mutants that escaped the effect of the protease. Domains are annotated according to a FoldSeek homolog search from the AlphaFold2 predicted structure [23,24], with CBM indicating carbohydrate binding module. Dark and light green mark the portion of the protein modeled in panels 2D and 2E, respectively. **d.** The C terminus of the protein mutated in phage Alon escapers is predicted by Alphafold2 to form hexamers (model ipTM = 0.79). The hexameric structure (dark green, amino acids 317-759) is structurally homologous (amino acids 589 through 716) to the tail tube protein of phage T4 (PDB code 5w5f, pink, one hexamer out of three hexamers presented in 5w5f is plotted for clarity). **e.** AlphaFold2 prediction of the N-terminus of the tail-associated protein (light green, amino acids 1 through 486) aligned with a homologous carbohydrate binding module (PDB code 3k4z, purple, amino acids 1 through 176).

To gain further insight into the mechanism underlying the protease effect on phage titers, we attempted to isolate phage mutants that escape the effect of the protease. Such escaper mutants were previously shown useful for predicting the specificity determinants of bacterial defense systems [21]. We collected phages from an infection experiment in liquid culture of *S. coelicolor* expressing the *Salinispora* protease and subjected them to two additional rounds of infection with bacteria expressing the *Salinispora* protease. Isolated escapers showed a partial or complete resistance to the antiphage effect of the *Salinispora* protease (Fig 2A). We have not observed such phenotype in single plaques isolated from phage survivors of infections in the presence of mutant *Salinispora* protease (Fig 2A).

We sequenced the genome of 5 isolated escaper phages that were partially or wholly resistant to the antiphage effect of the *Salinispora* protease. As a control we sequenced the genomes of 3 phages selected in an infection experiment in liquid culture of bacteria expressing mutant protease. All 5 escapers isolated on strains expressing the WT protease had a mutation in the same phage gene (Fig 2B, Fig 2C), but none of the control phages had mutations in that gene (Table S2 in S1 File). Four of the escaper phages had an additional deletion that was also observed in control phages selected on bacteria expressing a mutant protease, suggesting that this deletion is unrelated to the escape phenotype (S2 Table in S1 File).

To test the robustness of the immune evasion phenotype of the escaper phages, we repeated this process also for a related phage called Tapuz, which shows 70% homology to phage Alon over 42% of its nucleotide sequence [25], and which we also found sensitive to the effect of the *Salinispora* protease (Fig S2 in S1 File). Two Tapuz phage mutants that can escape protease-mediated defense were isolated, and in both cases these phages exhibited a mutation in an orthologous gene in phage Tapuz, which shares 46% sequence identity with the protein mutated in the phage Alon escapers (Fig 2B, 2C). One of the escaper phages (escaper no. 6) did not contain any other mutation except for the mutation in the indicated gene, showing that this mutation is sufficient for the escaper phenotype (Table S2 in S1 File).

We predicted the structure of the mutated phage protein using AlphaFold2 in monomer and multimer forms [23] and ran a structural homology search using Foldseek [24]. The C-terminus of the protein (amino acids 589–716 in phage Alon) showed structural homology to the tail tube protein of phage T4 [26], suggesting that this protein may function as a tail-associated protein in phage Alon. Similar to the T4 tail tube, the AlphaFold2 model for the C-terminus of the phage Alon tail tube predicted high confidence (ipTM = 0.79) hexameric structure of a similar diameter to the phage T4 hexameric protein (Fig 2D). The N-terminus of the predicted tail tube protein of phage Alon exhibits three consecutive domains that are structurally similar to each other as well as to carbohydrate binding modules (Fig 2E).

**Fig 3:**
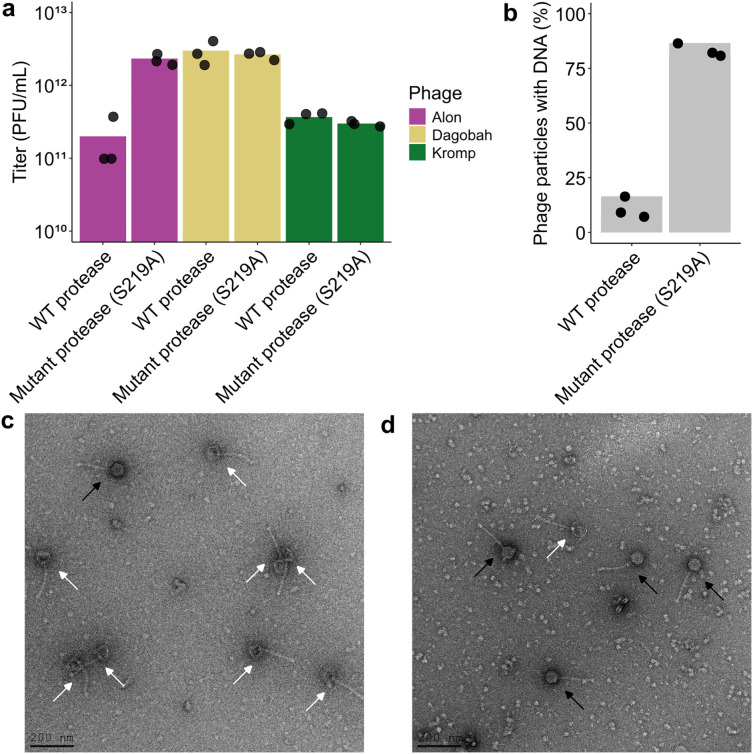
The *Salinispora* protease is associated with Phage Alon titer reduction and DNA loss. **a.** Incubation of phage particles with protease-containing supernatant results in titer loss. Phages Alon, Dagobah and Kromp were purified using CsCl gradient and incubated with concentrated supernatant from *S. coelicolor* cells expressing WT or catalytically inactive *Salinispora* protease. Phage titers shown are averages of three biological replicates with individual data points overlaid. **b.** *Salinispora* protease-dependent DNA-loss in phage Alon when incubated with concentrated supernatant from *S. coelicolor* cells expressing WT or catalytically inactive *Salinispora* protease. Particles were imaged under transmission electron microscopy and DNA content was recorded based on the observed density of the phage head. Graphs represent average percent of phages particles with DNA to total phage particles in counts of 300 particles per replicate. Shown is average of three replicates with individual data points overlaid. **c**. *Salinispora* protease-dependent DNA loss in phage Alon incubated with concentrated supernatant from *S. coelicolor* cells expressing WT *Salinispora* protease. Representative electron microscopy images of phages following treatment with concentrated supernatant from *S. coelicolor* expressing WT (**c**, left) or catalytically inactive S219A *Salinispora* protease (**d**, right). Black arrows point to phage particles with DNA and white arrows point to DNA-less “ghost” phage particles. Additional electron microscopy fields are presented in Fig S4 in S1 File.

Our data show that phages mutated in a predicted tail-associated protein can escape the effect of the *Salinispora* protease. To test the hypothesis that the antiphage effect of the *Salinispora* protease results from direct interaction with the phage particle, we incubated purified phage particles with concentrated supernatant from bacteria expressing the *Salinispora* protease. Phages incubated with the supernatant alone for one day showed 10-fold less infectivity as compared to phages incubated with a supernatant from bacteria expressing the mutated protease (Fig 3A). As with the antiphage effect in infection experiments in liquid culture, this bacteria-independent antiphage effect of the *Salinispora* protease was not observed in phages not homologous to phage Alon (Fig 3A).

We next imaged the particles of phage Alon by transmission electron microscopy. Following incubation of the phages with concentrated supernatant obtained from protease-expressing bacteria, we observed that 90% of the phages contained “empty” heads, suggesting that these phages lost their DNA content (Fig 3B-D). In contrast, Alon phages incubated with supernatant obtained from bacteria expressing the mutated protease mostly exhibited DNA-containing heads (Fig 3B-D, Fig S4 in S1 File). These data suggest that phages incubated with protease-containing supernatant tend to have their DNA ejected, explaining the reduction in infectivity for these phages.

## Discussion

In this study we demonstrate that a protease predicted to be secreted can affect the outcome of phage infection. When expressed in *S. coelicolor*, this *Salinispora*-originating protease is able to reduce infective titers of phages Alon and Tapuz after prolonged incubation, and we show that this activity is localized outside of the bacterial cell. The loss of phage infective titers was associated with increased ratios of phages that lost their DNA, suggesting that the protease causes loss of the DNA from affected phages. We furthermore found that phages mutated in a likely tail-associated protein can escape the effect of the protease.

One can envision several hypothetical mechanistic scenarios that can explain our observations. Under one hypothesis, cleavage of the structural protein in the phage tail leads to premature DNA ejection independent of the presence of bacteria, resulting in the observed DNA-less particles in the electron microscope. Within this hypothesis, escaper phages are resistant either to the direct proteolytic effect of the protease on the tail-associated protein or are able to maintain their DNA unejected even when their tail-associated protein is subjected to proteolysis.

An alternative hypothesis involving proteolysis is that the *Salinispora* protease cleaves off an extracellular portion of the bacterial protein used by the phage as a receptor. Within this hypothesis, the cleaved portion of the receptor protein is present in the supernatant derived from cells expressing the *Salinispora* protease, and the phage might bind it, thereby stimulating DNA ejection. Escaper phage mutants may have a reduced affinity to the phage receptor and require increased receptor concentration to lead to binding and DNA ejection. Phage T5, for example, is known to release its DNA *in vitro* in the presence of its bacterial receptor FhuA [27].

Our study has several limitations. First, it is possible that the primary role of the protease does not involve phage defense when expressed in its original *Salinispora* host. However, studies in the original bacteria encoding this protease are limited due to the slow growth rate of this marine bacterium in laboratory settings [28], as well as the absence of genetic systems and the lack of known phages for this host. While the protease we studied is located in variable genomic environments in *Salinispora* species, homologs of this protease in other bacteria do not tend to be enriched next to known defense systems in bacterial genomes, implying that this family of proteases have roles other than phage defense. It is therefore possible that the effect we observed when expressing this protease in *S. coelicolor* is a side effect of the real biological activity of the protease.

Another limitation of this study is that we worked with supernatants from protease-expressing cells rather than with a purified protease. While we demonstrated that supernatants contain the secreted protease, and that the antiphage activity we found in the supernatants is dependent on an intact protease active site, we cannot rule out that the activity we observed involves other factors. Unfortunately, we have not managed to purify this protease from *E. coli* cells. Future studies with purified proteases can examine whether the protease alone has a direct effect on premature DNA ejection from phage particles. In addition, future experiments with different growth media or expression conditions may lead to better expression outcomes.

A puzzling observation in our study is that phage Alon can readily infect and replicate on *S. coelicolor* cells even when these cells express the *Salinispora* protease (Fig S5 in S1 File). The reduction in infective titers is only observed after the phages, which already replicated, are further incubated with the cells (or the supernatant derived from these cells) for a prolonged period of two days. This can be explained in several ways. First, it is possible that the protease activity is very slow, and only affects the phages after long incubation. Second, it is possible that the antiphage activity necessitates another factor that is produced by *Streptomyces* cells only in a late developmental stage. It is known that *Streptomyces* species produce some secondary metabolites only after 2–3 days from spore germination [29], and in some cases even longer periods are required for the production of secondary metabolites [29,30].

Most bacterial defense systems investigated to date were heterologously expressed in *E. coli* or *B. subtilis* and originated from members of their respective phyla [1–3]. Recent studies showed that using expression hosts from the *Actinomycetota* phylum, and specifically *Streptomyces* bacteria, can lead to the discovery of new concepts in antiphage defense [31–34]. Our study is an additional demonstration of the benefit of using *Streptomyces* to study bacterial defenses. As *Actinomycetota* often live in complex ecological environments and exhibit multicellularity characteristics, it might make sense that these bacteria would involve extracellular antiphage activity as part of their defensive arsenals. Further studies with *Streptomyces* may find additional such extracellular mechanisms in the future.

## Materials and methods

Spore stocks of *S. coelicolor* and *Streptomyces lividans* strains were prepared as previously described [35]. Conjugation and genome integration of integrative plasmids into *S. coelicolor* was performed as previously described [36], with the following modification: pRK2013 [37] was used as the helper conjugation plasmid instead of pUB307. Phage infection experiments in liquid culture were performed in DNB MMC media, (Difco nutrient broth, supplemented with 10 mM MgCl_2_, 0.1 mM MnCl_2_ and 8 mM CaCl_2_) at 30°C in 15 mL culture tubes shaking at 350 rpm, unless specified otherwise. Phage sequence homology search was performed using blastn with the default search parameters [25]. Protein structure prediction from sequence was performed using AlphaFold2 [23]. Protein structural homolog search was performed using Foldseek [24]. Protein structures were visualized using PyMOL [38]. No permits were required for the described study, which complied with all relevant regulations.

### Phage isolation from soil

Phages Alon, Tapuz and Zayit were isolated from soil following previously reported protocols [31], with modifications as described hereby: In a 15 mL culture tube, *S. coelicolor* M145 was added at a concentration of 5 × 10^6^ CFU/mL to 5 mL of DNB with 0.5%(w/v) glucose and 4 mM CaCl_2_. 2 mL of soil were added as well and samples were incubated at 30°C overnight. Supernatant was collected by two rounds of centrifugation followed by filtration. The supernatant was serially diluted and plated on DNB to obtain single plaques as described below: 100 μL spore suspension of *S. coelicolor* M145 (10^8^ CFU/ mL) were added to 15 mL screw cap tubes. 5 mL of DNB MMC with 0.5%(w/v) agar at 50°C were then added and the resulting phage/spore suspension was mixed and plated on 100 X 15 mm plates with 20 mL DNB 2%(w/v) agar. The plates were incubated overnight at 30°C. Single plaques were picked with a pipette tip into 120 μL phage buffer (50 mM Tris pH 7.4, 100 mM MgCl_2_, 10 mM NaCl). Two more rounds of plating for single plaque and picking ensued and then phages were amplified as described below, under ‘phage stocks preparation from single plaques’, to obtain the phage stock. Phage DNA sequencing was performed as previously described [21].

### Phage stocks preparation from single plaques

To prepare phage stocks from single plaques, phage stocks were serially diluted, 20 μL into 180 μL, and added to a 100 μL spore suspension of *S. coelicolor* M145 (10^8^ CFU/ mL) in 15 mL screw cap tubes. 5 mL of DNB MMC with 0.5%(w/v) agar at 50°C were then added and the resulting phage/spore suspension was mixed and plated on 100 × 15 mm plates with 20 mL DNB 2%(w/v) agar. The plates were incubated overnight at 30°C followed by incubation at 25°C until single plaques were visible, if necessary. Single plaques were picked with a pipette tip into 120 μL phage buffer.

To obtain confluent lysis, single plaque suspensions in phage buffer were serially diluted, 20 μL into 180 μL, in phage buffer and added to 100 μL spore suspension of *S. coelicolor* M145 (10^8^ CFU/ mL) in 15 mL screw cap tubes. 5 mL of DNB MMC 0.5%(w/v) agar at 50°C were then added and the resulting phage/spore suspension was mixed and plated on 100 × 15 mm plates with 20 mL DNB 2%(w/v) agar.

Phage stocks were prepared from confluent lysis plates by scraping the 5 mL 0.5%(w/v) agar layer onto 6 mL DNB in 15 mL screw cap tubes, vortexing and shaking intermittently for 1 h at 25°C, followed by 15 min centrifugation at 3200 *g* at 4°C. The supernatant was then filtered through 0.2 μ filters and phage concentration was measured by plaque assay, as described below, under ‘phage titer measurement by plaque assay’.

### Infection experiment of *S. coelicolor* with phages in liquid culture

Spores of *S. coelicolor* encoding WT *Salinispora* protease, mutant S219A *Salinispora* protease or no *Salinispora* protease (Table S3 in S1 File, strains 5–7) were added in biological triplicates to 750 μL of DNB MMC in culture tubes of 15 mL at a concentration of 3 × 10^7^ CFU/mL, along with relevant phage in multiplicity of infection (MOI) of 0.1 and incubated for 45 h, unless otherwise indicated. Following incubation, phages were harvested by pelleting bacterial cells at 3200 g at 4°C for 30 min, followed by filtration through 0.2 μ cellulose acetate filters. Phage titer was then measured by plaque assay as described blow.

### Phage titer measurement by plaque assay

Phage stocks were serially 10 × diluted, 20 μL to 180 μL, in phage buffer, to obtain 10^0^-10^-7^ dilutions. A square petri dish of *S. coelicolor* M145 was prepared by mixing 30 mL of 50°C DNB MMC medium with 0.5%(w/v) agar with 10^6^ CFU of M145 spores and left to cool for 1 h. Onto that plate, 10 μL of each phage dilution were dropped, left to dry for 1 h and placed in 30°C overnight, followed by incubation at 25°C until single plaques were visible, if necessary. Phage titer was calculated by counting individual plaques in the highest concentration dilution where single plaques are countable and multiplying by the corresponding dilution factor.

### Supernatant harvest from *S. coelicolor* cultures

Spores of *S. coelicolor* encoding WT *Salinispora* protease, mutant S219A *Salinispora* protease or no *Salinispora* protease (Table S3 in S1 File, strains 5–7) were added in biological triplicates to 6 mL of DNB MMC at a concentration of 3 × 10^7^ CFU/mL, equally divided to 2 culture tubes of 15 mL (3 mL in each tube) and incubated for 46 h. Following incubation, supernatants from each two duplicate tubes were combined and harvested by pelleting bacterial cells at 3200 *g* at 4°C for 30 min, followed by filtration through 0.2 μ cellulose acetate filters. Larger molecules in the supernatant were then concentrated by transferring to Amicon® Ultra-4 10 KDa filters and centrifugation at 3200 *g* at 4°C for 60 min. Samples were then placed on ice until use for infection experiments in liquid culture and frozen at −20°C prior to supernatant analysis by protein MS.

### Supernatant analysis by protein MS

Supernatant samples were subjected to in-solution tryptic digestion following the S-trap protocol (Protifi) followed by a solid phase extraction cleanup step. The resulting peptides were analyzed using nanoflow liquid chromatography (nanoAcquity) coupled to high resolution, high mass accuracy mass spectrometry (Thermo Q-Exactive HFX). The samples were analyzed on the instrument separately in a random order in discovery mode. Raw data was processed with the MetaMorpheus v1.0.2 informatics platform. The data were searched with semi-tryptic parameters against a protein database containing the *S. coelicolor* protein database as downloaded from Uniprot [39] on August 2023, the WT and mutant *Salinispora* protease, and predicted phage Alon open reading frames larger than 100 amino acids extracted using NCBI ORFFinder [40], and a list of common lab contaminants.

### Infection experiments in liquid cultures with exogenously added supernatant

Spores of *S. coelicolor* M1146 not encoding WT *Salinispora* protease (Table S3 in S1 File, strain 2) were added to 750 μL of DNB MMC in 9 culture tubes of 15 mL at a concentration of 3 × 10^7^ CFU/mL, along with phage Alon at MOI = 0.1 and 10 μL of harvested supernatants from strains 5–7 (Table S3 in S1 File) in biological triplicates and incubated at 30°C for 48 h. Following incubation phages were harvested by pelleting bacterial cells at 3200 *g* at 4°C for 30 min, followed by filtration through 0.2 μ cellulose acetate filters. Phage titer was then measured by plaque assay.

### Selection for phages that escape the antiphage activity of the *Salinispora* protease

Three biological replicates of each phage (Alon or Tapuz) were amplified from single plaques on *S. coelicolor* M145. Each biological replicate of the phage was amplified on *S. coelicolor* Strains 5 and 6 (Table S3 in S1 File) expressing the WT *Salinispora* protease or the catalytically inactive S219A mutant, each in three biological replicates, as well. The phage amplification was set up similarly to the infection experiment of *S. coelicolor* with phages in liquid culture, but a higher volume and a variable incubation time was used, as described herby.

Round 1: Spores of *S. coelicolor* encoding WT *Salinispora* protease and mutant S219A *Salinispora* protease (Table S3 in S1 File, strains 5–6) were added in biological triplicates to 1.5 mL of DNB MMC in 6 culture tubes of 15 mL at a concentration of 3 × 10^7^ CFU/mL, along with phage Alon or phage Tapuz at MOI = 0.1 and incubated at 30°C for 39 h. Following incubation, phages were harvested by pelleting bacterial cells at 3200 *g* at 4°C for 30 min, followed by filtration through 0.2 μ cellulose acetate filters and phage titer was then measured by plaque assay.

Round 2: for phage Alon or phage Tapuz, three phage stocks that were amplified over Round 1 on *S. coelicolor* expressing WT *Salinispora* protease were added in 1/10 of the total volume into 1.5 mL of DNB MMC in 6 culture tubes (3 of *S. coelicolor* expressing WT *Salinispora* protease and 3 expressing mutant protease) at a concentration of 3 × 10^7^ CFU/mL. The amplification on *S. coelicolor* expressing WT *Salinispora* proteas was used for escaper isolation while the amplification over on *S. coelicolor* expressing mutant S219A *Salinispora* protease was used as a control to assess escaper phenotype evolution. MOI was varied depending on the phage stock and ranged between 0.001 and 0.1. As a control, one phage stock amplified over Round 1 on *S. coelicolor* expressing mutant *Salinispora* protease (S219A) was added in MOI 0.1 and 0.001 for phage Alon and Tapuz, respectively, to two tubes with spores of *S. coelicolor* expressing WT and S219A mutant *Salinispora* protease at a concentration of 3 × 10^7^ CFU/mL. All tubes were incubated at 30°C for 50 h. Following incubation, phages were harvested by pelleting bacterial cells at 3200 *g* at 4°C for 30 min, followed by filtration through 0.2 μ cellulose acetate filters and phage titer was then measured by plaque assay.

Round 3: for phage Alon or phage Tapuz, three phage stocks amplified over Round 2 on *S. coelicolor* expressing WT *Salinispora* protease were added in 0.1 and 0.01 MOI for phage Alon and Tapuz, respectively, into 1.5 mL of DNB MMC in 6 culture tubes (3 of *S. coelicolor* expressing WT *Salinispora* protease and 3 expressing mutant protease) at a concentration of 3 × 10^7^ CFU/mL. As a control, one phage stock amplified over Round 2 on *S. coelicolor* expressing mutant *Salinispora* protease (S219A) was added in MOI 0.1 to two tubes with spores of *S. coelicolor* expressing WT and S219A mutant *Salinispora* protease at a concentration of 3 × 10^7^ CFU/mL. All tubes were incubated at 30°C for 44 h. Following incubation, phages were harvested by pelleting bacterial cells at 3200 *g* at 4°C for 30 min, followed by filtration through 0.2 μ cellulose acetate filters and phage titer was then measured by plaque assay.

For each of Round 2 and Round 3, phages that were amplified in liquid culture on *S. coelicolor* expressing WT *Salinispora* protease were plated to obtain single plaques on *S. coelicolor* expressing WT *Salinispora* protease. Control phages that were amplified in liquid culture on *S. coelicolor* expressing S219A mutant protease were plated on *S. coelicolor* expressing S219A mutant *Salinispora* protease. Except for the bacterial strain expressing *Salinispora* protease, single plaque plating was as described above: phage stocks were serially diluted and added to a 100 μL spore suspension of *S. coelicolor* (expressing WT or S219A *Salinispora* protease at 10^8^ CFU/ mL) in 15 mL screw cap tubes. 5 mL of DNB MMC with 0.5% (w/v) agar at 50°C were then added and the resulting phage/spore suspension was mixed and plated on 100 × 15 mm plates with 20 mL DNB 2% (w/v) agar. The plates were incubated overnight at 30°C. Single plaques were picked with a pipette tip into 120 μL phage buffer.

Half of the single plaque suspension was used for infection in liquid culture with *S. coelicolor* expressing WT *Salinispora* protease and half was used for infection in liquid culture with *S. coelicolor* expressing S219A mutant *Salinispora* protease at a spore concentration of 3 × 10^7^ CFU/mL. Total volume in DNB MMC was 1.5 mL and incubation was for two days. Following incubation, phages were harvested by pelleting bacterial cells at 3200 *g* at 4°C for 30 min, followed by filtration through 0.2 μ cellulose acetate filters and phage titer was then measured by plaque assay. Phage DNA was harvested from single plaque thus amplified in liquid culture on *S. coelicolor* expressing WT protease if the titer was < l0x reduced compared with the same single plaque suspension amplified on *S. coelicolor* expressing mutant S219A *Salinispora* protease. Such escaper phenotype was markedly different than the > 100X titer reduction commonly observed for WT phages Alon and Tapuz (Fig S2 in S1 File). Phage DNA sequencing and mutation analysis was performed as previously reported [21].

### Phage purification by CsCl gradient

Phages Alon, Dagobah and Kromp were amplified on 3 plates of 150 × 15 mm dimensions each of *S. coelicolor* M145 to obtain 40 mL stocks of 1 × 10^11^ PFU/mL (Alon and Dagobah) and 2 × 10^10^ PFU/mL (Kromp). Phage purification was as previously reported [41], with modifications as described hereby. Phages were pelleted at 25,000 *g* at 4°C for 2 h. The pellet was resuspended with 0.5 mL of phage buffer and loaded on CsCl step gradients (2 ml of ρ = 1.3, 3 ml of ρ = 1.4, 3 ml of ρ = 1.5, 2 ml of ρ = 1.7; all in phage buffer) formed in open-top polyclear ultracentrifugation tubes (Seton Scientific). The gradients were centrifuged in an SW41 rotor (Beckman) at 25,000 rpm and 4°C for 2 h and the phage bands collected by needle side-puncture. Buffer exchange for the extracted phages was done using Amicon 4 mL centrifugal filters (10 kDa molecular weight cutoff) in steps reducing ionic strength. First, 3 M NaCl in phage buffer was added followed by 3200 g at 4°C for 15 min and the flowthrough was removed. 2 M NaCl in phage buffer was then added followed by 3200 g at 4°C for 15 min and the flowthrough was removed. 1 M NaCl in phage buffer was then added followed by 3200 g at 4°C for 15 min and the flowthrough was removed. Phage buffer was then added followed by 3200 *g* at 4°C for 15 min and the flowthrough was removed. Finally, phage buffer was added followed by 3200 *g* at 4°C for 60 min for a final purified phage volume of 100 μL.

### Purified phage incubation with concentrated supernatant

Purified phage, phage buffer and concentrated supernatant were mixed in PCR tubes as follows: 4 μL of purified phage (Alon, Dagobah or Kromp, purified as described in the section above discussing CsCl gradient), 2 μL of concentrated supernatant (from *S. coelicolor* expressing WT *Salinispora* protease or mutant S219A *Salinispora* protease, each in three biological replicates; harvested as described in the section titled ‘supernatant harvest from *S. coelicolor* cultures’) and 2 μL of phage buffer. Samples were incubated without shaking at the following temperature gradient: 3 h at 30°C, 3 h at 25°C, 3 h at 20°C, 3 h at 15°C, 7 h at 10°C and 7 h at 4°C. Phage titers were counted by plaque assay as described above. Complete phage particles with and without DNA were counted from electron microscopy images following negative staining as described below.

### Negative staining and electron microscopy imaging

For negative staining, formvar and carbon-coated copper grids (300 mesh, Electron Microscopy Sciences) were glow discharged in an Evactron CombiClean Decontaminator (XEI Scientific) for 2 min at 0.067 mbar (air) and 18 W. 3 μL of the phage sample was then directly applied to the grid, incubated for 1 min and blotted with filter paper (Whatman Grade 1). The grid was briefly washed on a drop of water and blotted with filter paper. Staining was performed by touching a 10 μL drop of 2% (w/v) uranyl acetate, followed by blotting with filter paper. This step was repeated once more. Then the grid was placed on a third 10 μL drop of 2% (w/v) uranyl acetate for 1 min. Finally, the grid was blotted with filter paper and air dried. Imaging was performed using a Tecnai Spirit (Bio Twin) transmission electron microscope (Thermo Fisher Scientific) at an accelerating voltage of 120 kV and a Gatan OneView camera.

## Supporting information

S1 File Figure S1. Mass spectrometry (MS) analysis indicates presence of the catalytically inactive protease mutant in supernatants. Figure S2. Titers of phages Alon and Tapuz following infection with *S. coelicolor* expressing the WT *Salinispora* protease (WT) or a catalytically inactive mutant of the protease. Figure S3. Phage Alon variants selected on bacteria expressing the WT *Salinispora* protease (Escaper) or catalytically inactive protease (Control). Figure S4. *Salinispora* protease-dependent DNA loss in phage Alon incubated with concentrated supernatant from *S. coelicolor* cells expressing WT *Salinispora* protease but not mutant protease. Figure S5. Time course experiment with phage Tapuz showing how phage titers change over time in an infection experiment of *S. coelicolor* encoding the WT *Salinispora* protease (red) or a negative control not encoding the protease (blue). Table S2. Phages used in this study. Table S3. Bacterial strains used in this study. Table S4. Plasmids used in this study.
(DOCX)

S2 File Plasmid map of 15_2517459620_Gib_in_pCAP03. (GBK)

S3 File Plasmid map of 15_2517459620_S219A_Gib_in_pCAP03. (GBK)

S4 File Plasmid map of 15_in_pCAP03. (GBK)

S5 File Plasmid map of 3_actinomycin-50-bp-homo-capture-vector-pCAP03. (GBK)

S6 File Figure 1c source data. (CSV)

S7 File Figure 1d source data. (CSV)

S8 File Figure 1e source data. (CSV)

S9 File Figure 1f source data. (CSV)

S10 File Figure 2a source data. (CSV)

S11 File Figure 3a source data. (CSV)

S12 File Figure 3b source data. (CSV)

S13 File Figure S1 source data. (CSV)

S14 File Figure S2 source data. (CSV)

S15 File Figure S3 source data. (CSV)

S16 File Figure S5 source data. (CSV)

S17 File Table S1. (XLSX)
