# Supplementary material for: Extracellular activity of a bacterial protease associated with reduced phage infectivity
Source: PLoS One. 2026 May 14;21(5):e0332566. doi: 10.1371/journal.pone.0332566 (PMC13175485; doi:10.1371/journal.pone.0332566)
Supplement: S1 File — Figure S1. Mass spectrometry (MS) analysis indicates presence of the catalytically inactive protease mutant in supernatants. Figure S2. Titers of phages Alon and Tapuz following infection with S. coelicolor expressing the WT Salinispora protease (WT) or a catalytically inactive mutant of the protease. Figure S3. Phage Alon variants selected on bacteria expressing the WT Salinispora protease (Escaper) or catalytically inactive protease (Control). Figure S4. Salinispora protease-dependent DNA loss in phage Alon incubated with concentrated supernatant from S. coelicolor cells expressing WT Salinispora protease but not mutant protease. Figure S5. Time course experiment with phage Tapuz showing how phage titers change over time in an infection experiment of S. coelicolor encoding the WT Salinispora protease (red) or a negative control not encoding the protease (blue). Table S2. Phages used in this study. Table S3. Bacterial strains used in this study. Table S4. Plasmids used in this study. (DOCX) [file pone.0332566.s001.docx]

Extracellular activity of a bacterial protease associated with reduced phage infectivity

Ehud Herbst^1^, Gael Rosen Blechman^1^, Taya Fedorenko^1^, Sarah Melamed^1^, Gil Amitai^1^ and Rotem Sorek^1^

^1^Department of Molecular Genetics, Weizmann Institute of Science, Rehovot, Israel

# Supplementary information

#
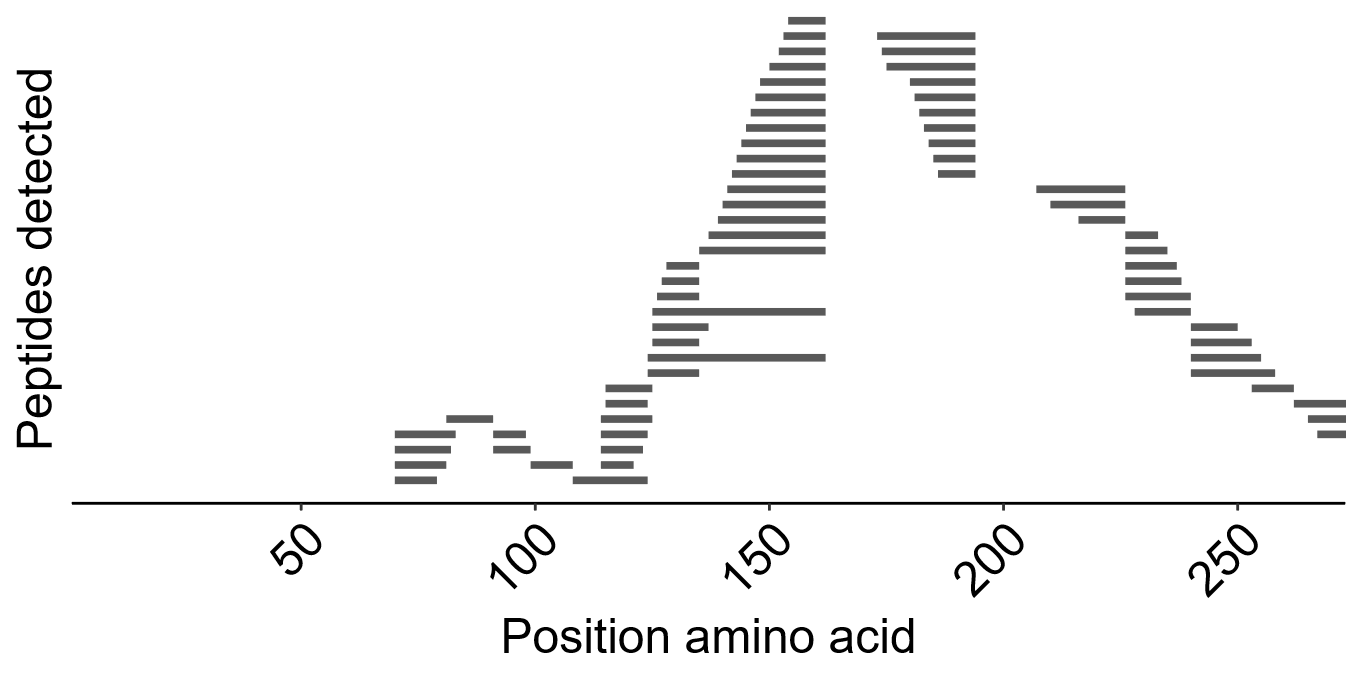
 Figure S1. Mass spectrometry (MS) analysis indicates presence of the catalytically inactive protease mutant in supernatants. Shown are non-redundant peptides (n=66) detected using MS. *S. coelicolor* expressing the catalytically inactive S219A *Salinispora* protease mutant was grown for 46 h, and the supernatant was harvested and filtered. Samples were digested with trypsin and were then subjected to protein MS.

**
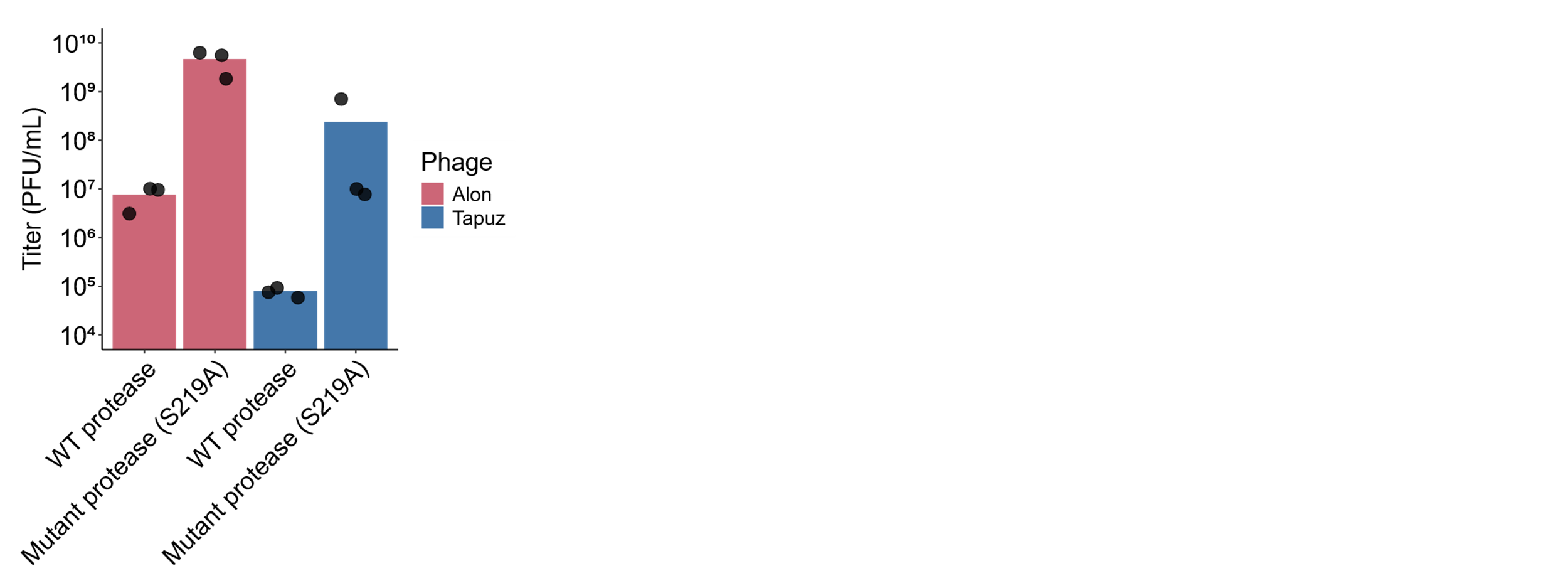
**

**Figure S2.** Titers of phages Alon and Tapuz following infection with *S. coelicolor* expressing the WT *Salinispora* protease (WT) or a catalytically inactive mutant of the protease. Phages were initially added at MOI=0.05, and harvested from the culture two days from the onset of infection. Bars show the averages of three biological replicates with individual data point overlaid.

**
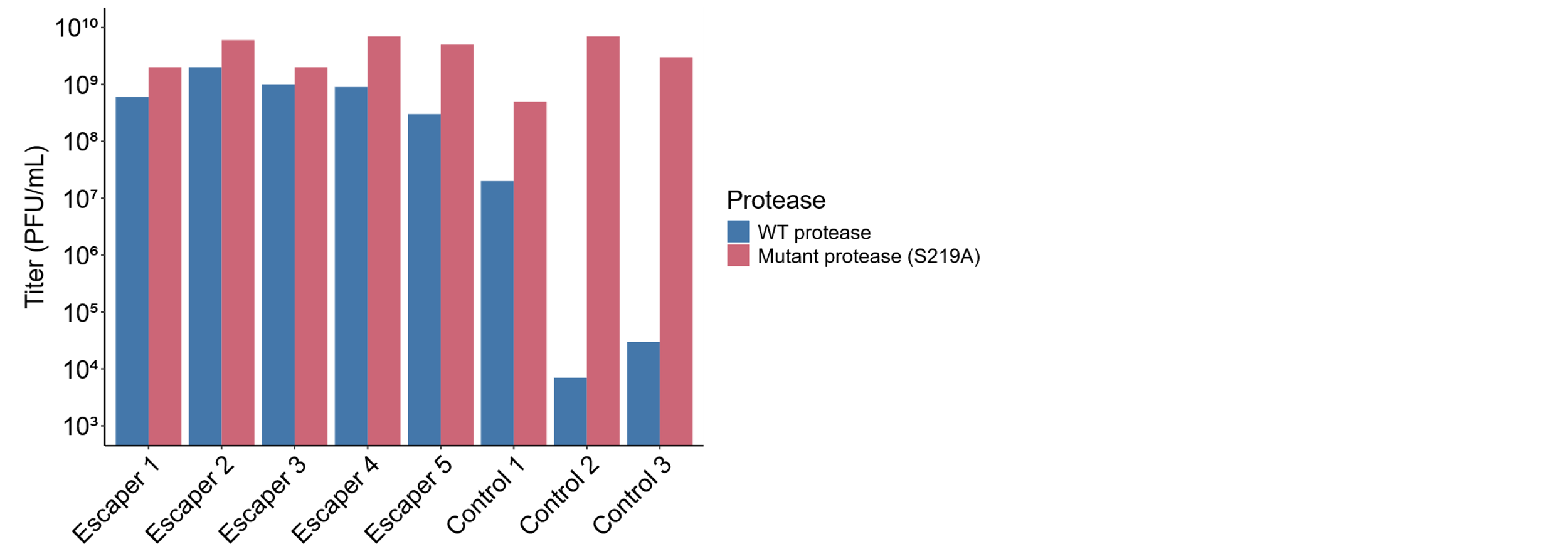
**

**Figure S3.** Phage Alon variants selected on bacteria expressing the WT *Salinispora* protease (Escaper) or catalytically inactive protease (Control). Shown data are results obtained for five escaper phages and three control phages. Genotypes for all mutants that were isolated and sequenced are presented in Table S2. Data for Escapers 2 and 4, and for Control phages 1 and 2, are also presented in Figure 2A.

**Figure S4.** *Salinispora* protease-dependent DNA loss in phage Alon incubated with concentrated supernatant from *S. coelicolor* cells expressing WT *Salinispora* protease but not mutant protease. Additional representative electron
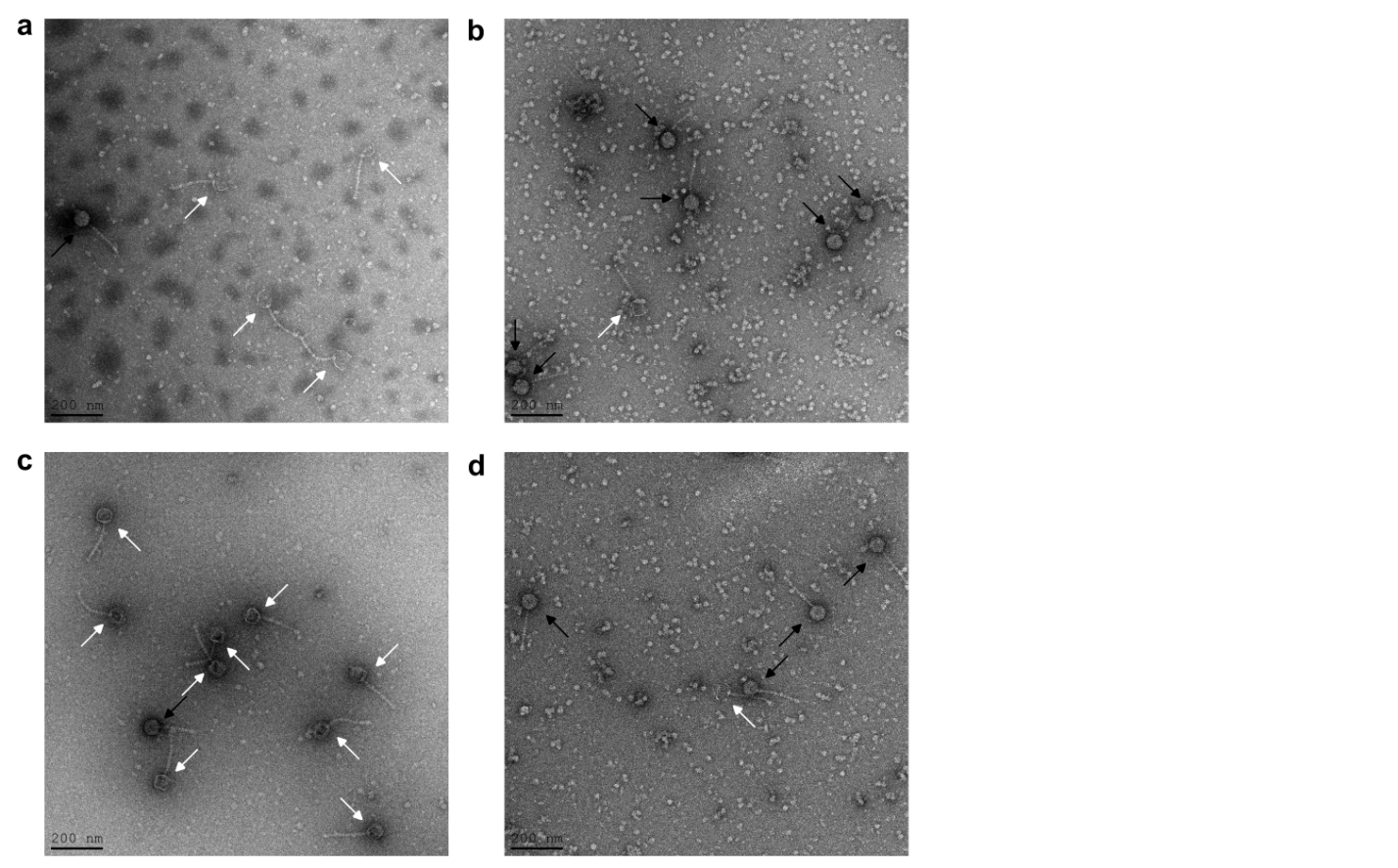
microscopy images of phages following treatment with concentrated supernatant from *S. coelicolor* expressing WT (**a, c**, left) or catalytically inactive S219A *Salinispora* protease (**b, d**, right). Black arrows point to phage particles with DNA and white arrows point to DNA-less phage “ghost” particles.

**
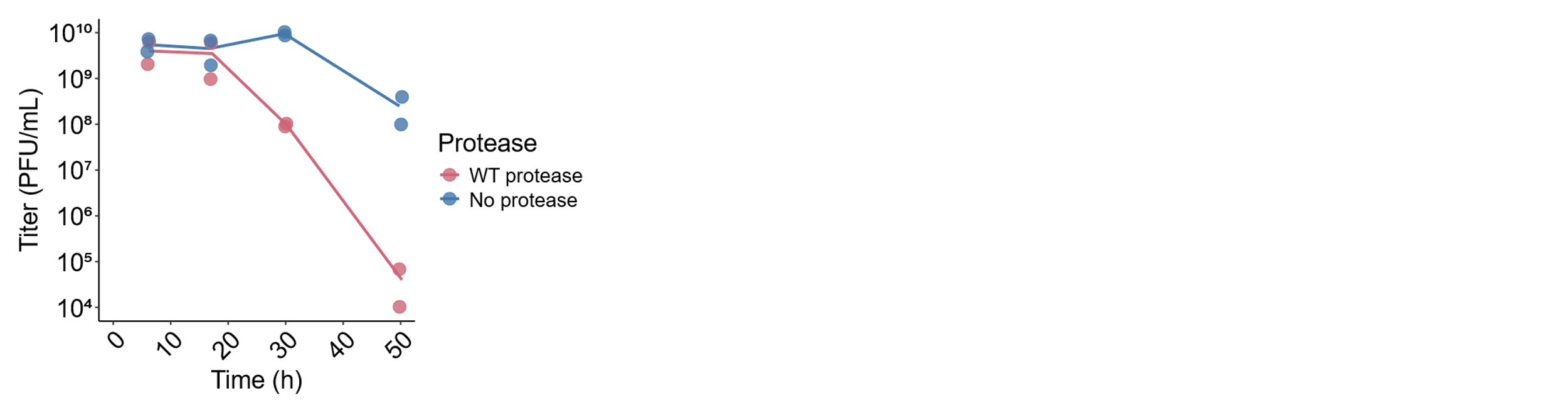
Figure S5.** Time course experiment with phage Tapuz showing how phage titers change over time in an infection experiment of *S. coelicolor* encoding the WT *Salinispora* protease (red) or a negative control not encoding the protease (blue). One of the two strains encoding the protease (red), encodes it within a 1.4 kb genomic fragment derived from *S. mooreana* (NCBI Reference sequence: NZ_KB900614.1; range: 4542153 to 4543551), while the other encodes it within a larger genomic fragment of 15.2 kb taken from the same strain (NCBI Reference sequence: NZ_KB900614.1; range: 4542146 to 4557358)*.* One of the control strains (those not encoding the protease, blue), encodes an apramycin resistance gene instead of the genomic fragment containing the protease (pCAP03-acc(3)IV, Addgene 69862; Range: 700 to 2075), while the other encodes genomic fragments from *Streptomyces anulatus* instead of the genomic fragment containing the protease (GenBank: HM038106.1; ranges: 576 to 625 and 47697 to 47746, separated from each other by a PmeI restriction site). Phages were initially added at a concentration of 3×10^5^ PFU/mL, MOI=0.01 and harvested from the culture after 6 h, 17 h, 30 h and 50 h from the onset of infection. Lines show the averages of two strains encoding or not encoding the protease with individual data point overlaid.

**Table S2.** Phages used in this study

| Phage number | Escaper number | Phage name | Host bacteria amplified on | Mutations (escaper phages) | Comments | Source |
| --- | --- | --- | --- | --- | --- | --- |
| 1 |  | Tapuz | M145 |  |  | This study |
| 2 |  | Zayit | M145 |  |  | This study |
| 3 |  | Alon | M145 |  |  | This study |
| 4 |  | Boomer | M145 |  |  | PhagesDB |
| 5 |  | Manuel | *S. lividans* 1326 |  |  | PhagesDB |
| 6 |  | EhyElimayoE | *S. lividans* 1326 |  |  | PhagesDB |
| 7 |  | Dagobah | M145 |  |  | DSMZ |
| 8 |  | Kromp | M145 |  |  | PhagesDB |
| 9 | 1 | Alon D430E | 5) WT protease | C→A in position 14560 and Δ115 bp in 33665 | Escaper mutant in tail-associated protein | This study |
| 10 | 2 | Alon T388P | 5) WT protease | A→C in position 14432 and Δ115 bp in 33665 | Escaper mutant tail-associated protein | This study |
| 11 | 3 | Alon N134K | 5) WT protease | C→A in position 13672 and Δ115 bp in 33665 | Escaper mutant in tail-associated protein | This study |
| 12 | 4 | Alon T388P | 5) WT protease | A→C in position 14432 and Δ115 bp in 33665 | Escaper mutant in tail-associated protein | This study |
| 13 | 5 | Alon A493V | 5) WT protease | C→T in position 14748  and C→G in 33731 | Escaper mutant in tail-associated protein | This study |
| 14 |  | Alon control escaper | 6) Mutant protease | Δ115 bp in position 33665 | Control escaper isolated on mutant S219A protease | This study |
| 15 |  | Alon control escaper | 6) mutant protease | Δ115 bp in position 33665 | Control escaper isolated on mutant S219A protease | This study |
| 16 |  | Alon control escaper | 6) Mutant protease | Δ115 bp in position 33665 | Control escaper isolated on mutant S219A protease | This study |
| 17 | 6 | Tapuz I420T | 5) WT protease | T→C in position 13964 | Escaper mutant in tail-associated protein | This study |
| 18 | 7 | Tapuz I420T | 5) WT protease | T→C in position 13964 and T→C in 28891 | Escaper mutant in tail-associated protein | This study |
| 19 |  | Tapuz control escaper | 6) Mutant protease | none | Control escaper isolated on mutant S219A protease | This study |
| 20 |  | Tapuz control escaper | 6) Mutant protease | none | Control escaper isolated on mutant S219A protease | This study |

**Table S3.** Bacterial strains used in this study

| Strain number | Strain description | Parent strain | Integrative plasmid | Source |
| --- | --- | --- | --- | --- |
| 1 | *S. coelicolor* M145 |  | - | Mervyn J. Bibb, John Innes Centre |
| 2 | *S. coelicolor* M1146 |  | - | Mervyn J. Bibb, John Innes Centre |
| 3 | *S. lividans* 1326 |  | - | DSMZ 46482 |
| 4 | *E. coli* ET12567 |  | - | ATCC BAA-525 |
| 5 | WT *Salinispora* protease in M1146 | M1146 | 15_2517459620_Gib_in_pCAP03 | This study |
| 6 | Mutant *Salinispora* protease S219A in M1146 | M1146 | 15_2517459620_S219A_Gib_in_pCAP03 | This study |
| 7 | pCAP03 (addgene) in M1146 | M1146 | pCAP03 | This study |
| 8 | WT *Salinispora* protease in M1146 within a larger *Salinispora* genome context (15.2 kb) | M1146 | 15_in_pCAP03 | This study |
| 9 | 0.1 kb genome fragment from *S. anulatus in* M1146 | M1146 | 3_actinomycin-50-bp-homo-capture-vector-pCAP03 | This study |

**Table S4.** Plasmids used in this study

| Plasmid number | Plasmid name | Plasmid description | Host strain | Source |
| --- | --- | --- | --- | --- |
| 1 | pRK2013 |  | ET12567 | DSMZ 5599 |
| 2 | pCAP03 |  | ET12567 | Bradley Moore, UCSD |
| 3 | 15_2517459620_Gib_in_pCAP03 | WT Salinispora protease in integrative plasmid pCAP03 | ET12567 | This study |
| 4 | 15_2517459620_S219A_Gib_in_pCAP03 | mutant Salinispora protease S219A in integrative plasmid pCAP03 | ET12567 | This study |
| 5 | 15_in_pCAP03 | WT *Salinispora* protease within a wider *S. mooreana* genomic context (15.2 kb) in integrative plasmid pCAP03 | ET12567 | This study |
| 6 | 3_actinomycin-50-bp-homo-capture-vector-pCAP03 | Acceptor vector for the actinomycin pathway containing 50 bp homology to the edges of the pathway in *S. anulatus* in integrative plasmid pCAP03 | ET12567 | This study |
